# A pilot clinical trial testing mutant von Hippel-Lindau peptide as a novel immune therapy in metastatic Renal Cell Carcinoma

**DOI:** 10.1186/1479-5876-8-8

**Published:** 2010-01-28

**Authors:** Osama E Rahma, Ed Ashtar, Ramy Ibrahim, Antoun Toubaji, Barry Gause, Vincent E Herrin, W Marston Linehan, Seth M Steinberg, Frank Grollman, George Grimes, Sarah A Bernstein, Jay A Berzofsky, Samir N Khleif

**Affiliations:** 1 Vaccine Branch, NCI, NIH, Bethesda, MD, USA; 2 Medical Oncology Clinical Research Unit (MOCRU) at the NNMC, Bethesda, MD, USA; 3 Department of Hematology Oncology, National Naval Medical Center, Bethesda, MD, USA; 4 Urologic Oncology Branch, Center for Cancer Research, NCI, NIH, Bethesda, MD, USA; 5 Biostatistics and Data Management Section, CCR, NCI, NIH, Bethesda, MD, USA; 6 Department of Pharmacy, Clinical Center, NIH, Bethesda, MD, USA

## Abstract

**Background:**

Due to the lack of specific tumor antigens, the majority of tested cancer vaccines for renal cell carcinoma (RCC) are based on tumor cell lysate. The identification of the *von Hippel-Lindau *(*VHL*) gene mutations in RCC patients provided the potential for developing a novel targeted vaccine for RCC. In this pilot study, we tested the feasibility of vaccinating advanced RCC patients with the corresponding mutant VHL peptides.

**Methods:**

Six patients with advanced RCC and mutated *VHL *genes were vaccinated with the relevant VHL peptides. Patients were injected with the peptide mixed with Montanide subcutaneously (SQ) every 4 weeks until disease progression or until the utilization of all available peptide stock.

**Results:**

Four out of five evaluable patients (80%) generated specific immune responses against the corresponding mutant VHL peptides. The vaccine was well tolerated. No grade III or IV toxicities occurred. The median overall survival (OS) and median progression-free survival (PFS) were 30.5 and 6.5 months, respectively.

**Conclusions:**

The vaccine demonstrated safety and proved efficacy in generating specific immune response to the mutant VHL peptide. Despite the fact that the preparation of these custom-made vaccines is time consuming, the utilization of VHL as a vaccine target presents a promising approach because of the lack of other specific targets for RCC. Accordingly, developing mutant VHL peptides as vaccines for RCC warrants further investigation in larger trials. Trial registration: 98C0139

## Background

Renal cell carcinoma comprises the majority of malignant kidney tumors. It is relatively rare in the United States but its incidence has continued to rise since 1975 [1,2]. The lifetime risk of developing RCC is 1 in 11,000 [3]. Earlier detection and treatment of smaller renal tumors has not significantly reduced the mortality rate and about one-third of patients still present with metastatic disease [4]. Indeed, the mortality rate has continued to rise, which necessitates looking for a better therapeutic strategy [5,6].

RCC is one of the most resistant forms of cancers to both radiation and chemotherapy. Recently, the multitargeted tyrosine kinase inhibitors Sorafenib and Sunitinib have shown 10% and 34-44% objective response rates, respectively, in metastatic RCC [7-9]. Accordingly, we are still in need of novel and successful therapeutic approaches to RCC.

Clear cell renal carcinoma (CCRC) is the most common histological subtype of RCC and accounts for about 70% of cases [10]. This tumor is often regarded as immunogenic based on the observation of a 4% spontaneous regression in metastatic lesions [11-13], the abundant presence of tumor infiltrating lymphocytes (TIL) in tumor specimens, and the well-documented responses to some immuno-cytokines (Interleukin-2 [IL-2] and Interferon-α [IFN-α]) and vaccine therapy [14]. IL-2 and IFN-α have shown some efficacy in the metastatic setting, with response rates of 12-20% [15-17]. Studies of other cytokines, dendritic cell-based vaccines, and adoptive immunotherapy with TILs or lymphokine activated killer (LAK) cells have shown some minor benefit [18-20]. It has been shown that patients who are able to generate specific cytotoxic T cells (CTLs) against tumors show better prognosis [21,22]. In addition, we and others have demonstrated in previous clinical trials that vaccination with peptides from different cancers produces specific immunological responses (specific CTLs) in the corresponding cancers [23-27].

One obstacle to developing a renal cancer vaccine was to identify an RCC tumor-specific antigen [28]. Most RCC vaccine trials have employed unfractionated antigens derived from the tumor cells, with the goal of eliciting specific T-cell responses against multiple undefined antigens expressed by the tumor [28-34]. More than 60% of patients with sporadic RCC possess a detectable somatic mutation in the *von Hippel-Lindau *(*VHL*) gene [35,36]. Somatic mutations in *VHL *have been linked to the development of sporadic CCRC and hemangioblastomas. Most of these mutations are frameshift and the rest are missense, nonsense, or stop mutations [37-39]. Other mutated oncoproteins such as Ras and p53 have been previously explored as targets for vaccine therapy in humans. We and others have found these antigens safe and able to induce specific T cells against the mutant but not the wild antigens [27,40-42]. Accordingly, mutated *VHL *represents a novel potential target for clear cell RCC.

In this pilot study, we present our experience using the mutated VHL peptides as a vaccine for metastatic RCC. We show that the use of mutant VHL peptides for targeted vaccine therapy is feasible, safe, and capable of generating specific immunological responses, which provides incentive for further exploration in the management of advanced RCC.

## Methods

### Patients and eligibility criteria

Patients with locally advanced, recurrent, progressive, or metastatic RCC were enrolled in this pilot trial. All patients enrolled in the trial met the protocol eligibility criteria, including: histologically proven CCRC; tumors expressing mutated *VHL *gene resulting in a new amino acid sequence; lack of available standard systemic treatment; Eastern Cooperative Oncology Group (ECOG) performance status of 0 or 1; and a life expectancy of more than 3 months. Main exclusion criteria included: evidence of brain metastasis; history of autoimmune disease; history of other malignancies except basal cell carcinoma of the skin; and pregnancy. The study protocol was approved by the Institutional Review Boards of the National Cancer Institute (NCI) and the National Naval Medical Center (NNMC), Bethesda, Maryland. Written informed consent was obtained from all patients. The study was in compliance with the Helsinki Declaration.

### Vaccine preparation

All peptides were custom-designed based on the patient's own tumor *VHL *mutation and the potential binding affinity of the amino acid motif spanning the mutation to the patient's HLA (Table 1 and 2). Peptides were designed based on the predicted binding affinity using the BIMAS program http://bimas.cit.nih.gov/molbio/hla_bind/. In case of a single residue point mutation (peptides 3 and 4), the mutation was placed in the center and 8 residues were included on each side, so that every 9-mer containing the mutation would be included in the peptide, to cover most possible epitopes that included the mutation. In the case of peptide 2, a shorter version of that peptide was used to avoid residues that flanked the mutation and lead to solubility problem such as a second Cysteine (C) on the n terminus, which would lead to cross-linking of peptides and aggregation. Peptides 1 and 6 were frame shift mutations, creating totally novel sequences, so as much length as possible was used until reaching a stop codon, or having to avoid some residues such as Cysteine (C), as outline above. The same concept applied to peptide 5, in which the frame shift ORF ended with Arginine (R). To have enough length, the sequence was extended to the left by 8 of the wild type residues (unmutated); so that every 9-mer would contain at least one of the abnormal frame shift residues and thus no epitope in the peptide would be contained in the wild type sequence. Peptides were synthesized under GLP conditions using an automated synthesizer (Multiple Peptide Systems, San Diego, CA) and standard solid-phase chemistry. The peptides were packaged in vials by the National Institutes of Health (NIH) Clinical Center's Pharmacy. Safety, identity, and stability assays were conducted by the NIH Clinical Center Pharmaceutical Development Service (PDS). Assay results for each lot were submitted to the Cancer Therapeutic Evaluation Program (CTEP) Biological Drug Quality Assurance Committee for review and approval prior to human use. One hundred microliters of the patient dosage were re-analyzed by HPLC for purity and quantity of peptide, and sequenced by automated sequenator to confirm identity. Immediately prior to vaccination, 1000 μg of the mutant VHL peptide in 0.7 mL of normal saline were emulsified in 1:1 ratio with the adjuvant "Montanide ISA-51" (Seppic, Inc., Fairfield, NJ).

**Table 1:** VHL peptides used for vaccinations (corresponding mutant part of peptide underlined)

Patient	Mutant VHL peptide
1	YHTASVYSERAM
2	CLQVARSLVK
3	PGTGRRIHIYRGHLWL
4	RRIHSYRGDLWLFRDA
5	MEAGRPRPCCAR
6	RLALQRCRDTRWA

### Treatment and vaccination schedule

Eligible patients received a dose of 1000 μg of the emulsified corresponding mutant VHL peptide and "Montanide ISA-51." Half of the total volume of the vaccine (0.7 mL) was administered subcutaneously over each deltoid muscle. Patients were observed for 1 hour in the outpatient clinic to assess for any allergic reaction. Vaccinations were repeated every 4 weeks until disease progression or until the utilization of all available stock of the peptide.

### Immunologic monitoring

Prior to the first vaccination, patients were apheresed to obtain 1 × 10^9 ^peripheral blood mononuclear cells (PBMC). This procedure was repeated every-other cycle. In the other cycles a 100 mL of whole blood was collected by phlebotomy to obtain 1 × 10^7 ^PBMCs. Lymphocytes obtained by apheresis were frozen and saved for future immunologic testing. An automated Ficoll-hypaque density gradient separation was used to obtain the appropriate cell types for immunological assays. The IFN-γ ELISPOT assay was used to quantify mutated VHL peptide-specific CTLs.

#### DC preparation used to generate DC for the ELISPOT assay

Dendritic cells (DCs) for use in the ELISPOT assays were obtained by culturing autologous monocytes in Granulocyte-macrophage colony-stimulating factor (GM-CSF) and IL-4 according to widely established procedures. Briefly, frozen PBMCs were thawed and rested for 2 hours, followed by incubation in plastic flasks for 2 hours. The nonadherent cells were then washed away and the remaining adherent cells were cultured in 10% fetal bovine serum (FBS) DC medium containing 100 IU/mL GM-CSF (Leukine Sargramostim, Bayer HealthCare Pharmaceuticals, Seattle, WA) and 50 ng/mL IL-4 (PeproTech, Inc., Rocky Hill, NJ) for 6 days at 37°C. Cultures were fed at day 3-4 by removing one-half of the culture volume and adding an equal volume of fresh media containing sufficient GM-CSF and IL-4 for the entire culture volume. DCs were harvested on day 6, pulsed with antigen for 4 hours, and then matured overnight with 5 ng/mL Lipopolysaccharide (LPS). On day 7, DCs were harvested, washed, and the cell suspension volume adjusted for use in the ELISPOT assay.

#### ELISPOT assay

All ELISPOT assays were performed at NCI Frederick (CLIA certified lab). The ELISPOT assay using autologous antigen-pulsed DCs was validated and approved by the NIH Vaccine Oversight Committee. Two frozen normal donor controls with known responsive values were run with each assay to assure quality control of the assay results. ELISPOT assay was performed on freshly thawed PBMCs with no *in vitro *expansion cultures or cytokine addition. Autologous monocyte-derived dendritic cells (DCs) pulsed with antigen and matured with Lipopolysaccharide (LPS) overnight were used as the antigen presenting cells (APC). Briefly, the day before assay setup, 96-well polyvinylidene fluoride (PVDF) membrane, HTS opaque plates (Millipore, Billerica, Massachusetts, MSIPS40W10) were coated overnight with capture antibody, anti-human IFN-γ (10 μg/mL) in DPBS (αIFN-γ capture antibody, 1 mg/mL Mabtech, Cat# 3420-3-1000) at room temperature. Patient dendritic cells were harvested and were either pulsed with the patient's specific mutant VHL at 50 μg/mL, the irrelevant peptide TAX (LLFGYPVYV, an HLA-A2 binding peptide) at 3 μg/mL, or no peptide for 4 hours and then matured overnight with LPS at 37°C. Antibody-coated plates were washed the next day and blocked with 5% HuAB ELISPOT medium at 37°C for approximately 2 hours; 3 × 10^5 ^freshly thawed and 2-hour rested patients' PBMCs and 3 × 10^4 ^pulsed autologous DCs were used per well. The plates were incubated for 18-20 hours at 37°C. The next day, the plates were manually washed six times with DPBS, 0.05% Tween 20, followed by a 2-hour incubation at room temperature with a 1:2000 dilution of the biotinylated secondary antibody, anti-human IFN-γ, (1 mg/mL Mabtech, Cincinnati, OH, Cat# 3420-6-1000). After incubation and four washes to remove excess antibody, a 1:3000 dilution of streptavidin alkaline phosphatase (Mabtech, Cincinnati, OH, Cat#3310-10) was added to each well for 1 hour followed by 4 manual washes. Finally, The BCIP/NPT substrate, 100 ul/well, (KPL, Gaithersburg, Maryland, Cat# 50-81-08) was added and the reaction was stopped incubating in distilled water for 7-10 minutes, resulting in the development of spots. Plates were dried overnight and the spots were visualized and counted using the ImmunoSpot Imaging Analyzer system (Cellular Technology Ltd., Cleveland, OH). The results were calculated as: total number of experimental spots with DC = (PBMC + pulsed DC) - (PBMC + nonpulsed DC). From each patient, postvaccination PBMCs were compared to prevaccination as a baseline. A positive ELISPOT result for the patient was defined as a total number of experimental spots in the postvaccination sample of more than twofold above the total spots in the prevaccination sample.

#### Regulatory T cells (T regs)

Cryopreserved PBMCs were thawed rapidly at 37°C. The cells were transferred into 15 mL conical tubes (Corning, Lowell, MA) and diluted to 10 mL by dropwise addition of RPMI medium containing 20% FBS. The cells were pelleted by low-speed centrifugation at 250 xg for 10 min at 25°C. Supernatants were discarded and cell pellets resuspended in 5 mL of Dulbecco's phosphate buffered saline (D-PBS) containing 2% huAB serum to block cell surface Fc receptors. The samples were mixed briefly and incubated on ice for 15 minutes. Following incubation the cells were pelleted by centrifugation as described before, washed two times with D-PBS containing 2% bovine serum albumin (BSA; D-PBS/2% BSA) and resuspended in 1 mL of D-PBS/2% BSA. The cells were counted in a Coulter counter and adjusted to a final concentration of 10 × 10^6^/mL in D-PBS/2% BSA. The cells (1 × 10^6^/tube) were stained for surface markers (CD25, CD3, and CD4) for 20 minutes at room temperature (RT) in the dark and washed two times with D-PBS/2% BSA.

Intracellular staining for FoxP3 was carried out using human FoxP3 buffer prepared as described by the manufacturer (BD BioSciences, San Jose, CA). Briefly, following staining of surface antigens, cells were resuspended in 2 mL of fixing solution (buffer A) and incubated for 10 minutes at RT in the dark. Cells were washed two times with PBS/2% BSA, resuspended in 0.5 mL permeabilization solution (buffer C) and incubated for 30 minutes at RT in the dark. Cells were washed two times in PBS/2% BSA and stained with anti-human FoxP3 antibody for 30 minutes at RT in the dark. Cells were then washed two times and resuspended in 0.5 mL of PBS/2% BSA for four-color flow cytometric analysis using the FACSCanto cytometer (BD biosciences, San Jose, CA) running FACS Diva acquisition software (version 6.0). Each assay contained a parallel set of cells stained with relevant isotype controls (Alexa Fluor 488 IgG1 and PE IgG1).

Flow cytometric data analysis was carried out using FlowJo Software. T cells were identified by plotting CD3 by side scatter. CD4^+ ^T cells were identified by further gating the CD3^+ ^subset by forward and side scatter and by CD4. The regulatory CD4^+ ^T cell subset was identified by plotting CD25 versus FoxP3 with the quadstat setting determined based on the isotype control tube. The quadrant markers of the CD25 versus FoxP3 dot plot were set based on the isotype controls. In each case the pre and post samples were tested side by side in the same experiment and were done from frozen samples. This testing strategy was used to minimize variability from day to day in staining or thawing. The samples were tested in 4 independent setups over 3 days. We have included 2 internal controls in each experiment, one of those being a frozen leukapheresis sample that has been included in each test run as a measure of interassay reproducibility. In the limited number of assays we have performed using that control, the interassay CV% has been 33% (range of 3.4 to 9.4% for CD25/FoxP3+). Eliminating the outlier value of 9.4% reduces the CV to 15%.

### Clinical monitoring

Patients were evaluated for toxicity and tumor response during treatment and up to 2 years after the last vaccination. Physical examination and blood profiling were performed prior to each vaccination. Tumor response was assessed by the appropriate imaging technique, according to RECIST criteria, at baseline, then following every two vaccinations during therapy and every 3 months during follow-up. Disease progression was defined as the appearance of new lesions and/or 25% increase of measurable lesions as evident by CT scan. Once patients had progressed, follow-up was not required except to document late toxicities and death. Adverse events/toxicities were defined and graded according to the NCI Common Toxicity Criteria. Patients were taken off study in the case of disease progression or deterioration in performance status.

## Results

### Patient characteristics

Six patients with locally advanced, recurrent, progressive, or metastatic RCC were enrolled in this pilot trial. These patients had no available standard treatment or refused to receive one at the time of enrollment. Characteristics of the treated patients are summarized in Tables 2 and 3. All patients included in the trial had a somatic mutation of the *VHL *gene (Table 2). These mutations were single amino acid substitutions in three patients (patients 2, 3, and 4), while patients 1 and 5 had nucleotide deletion and patient 6 had nucleotide insertion resulting in frameshift mutations leading to the development of novel amino acid sequences. The patients had different HLA alleles, as shown in Table 2.

**Table 2:** *VHL *mutations and HLA types in vaccinated patients

Pt	DNA mutation	Protein mutation	HLA-A	HLA-B	HLA-DR	HLA-DQ
1	del TT 443-444	148 Phe-Cys fsX25	02	15, 40	04, 13	03, 06
2	T-C 497	166 Val-Ala	02,11	3701, 4001	1001, 13	0501, 06
3	G-T 332	111 Ser-Ile	03, 29	14, 35	01, 13	05, 06
4	C-G 343	115 His-Asp	02	07, 40	1302, 1501	ND
5	del C 183	62 Val-Cys fsX5	03,29	35, 44	01, 13	0501, 06
6	ins C 346-347	116 Leu-Pro fsX16	02,31	40, 51	0404, 11	0301, 0302

Abbreviations: del = deletion; fs = frameshift; X = stop codon; ins = insertion.

Patient 1 had a deletion of a thymine at two nucleotides (443 and 444) that resulted in a predicted frameshift starting at codon 148 with a phenylalanine to cysteine amino acid change, extending for 23 more codons, and ending with a premature stop codon at position 172. Patient 2 had a mutation at nucleotide number 497 resulting in a change from thymine to cytosine which led to a substitution in valine to alanine at position 166. Patient 3 had a mutation at nucleotide number 332 resulting in a change from guanine to thymine that led to a substitution in serine to isoleucine at position 111. Patient 4 had a mutation at nucleotide number 343 resulting in a change from cytosine to guanine which led to a substitution from histidine to aspartic acid at position 115. Patient 5 had a deletion of a cytosine at nucleotide number 183 that resulted in a predicted frameshift starting at codon 62 with a valine to cysteine amino acid change, extending for 3 more codons, and ending with a premature stop codon at position 66. Patient 6 had an insertion of a cytosine between two nucleotides (346 and 347) that resulted in a predicted frameshift starting at codon 116 with a leucine to proline amino acid change, extending for 14 more codons, and ending with a premature stop codon at position 131.

Of the six patients enrolled in the trial, five were male and one was female (patient 2). Patients had an average age of 62 years, with an ECOG performance status of (0) in three patients (patients 2, 3, and 6) and (1) in three patients (patients 1, 4, and 5; Table 3). All patients were pretreated with multiple conventional therapies prior to enrollment on the protocol. Radical nephrectomy was performed in all patients and surgical resection of the metastasis was performed in all patients except patient 4. Three patients received cytokines: patient 3 received low-dose IL-2 and IFN-α for 6 months as an adjuvant therapy; patient 4 received IFN-α for lung metastasis, and patient 5 received high-dose IL-2 for metastatic mediastinal lymphadenopathy followed by radical lymph node dissection and radiation therapy to the mediastinum. Radiofrequency ablation for lung metastases was performed twice in patient 6. Three patients (patients 2, 3, and 5) had no detectable disease on enrollment and the other three patients (patients 1, 4, and 6) had distant metastases (Table 3).

**Table 3:** Patient characteristics of the study population

Pt	Age	Gender	PS	Stage at diagnosis	Prevaccination therapy	Extent of disease at first vaccination
1	61	M	1	II	SX2	Lung and mediastinal LN metastasis
2	66	F	0	III	SX2	NED
3	40	M	0	III	SX3, IFN-α, IL-2	NED
4	71	M	1	IV	SX1, IFN-α	Lung and abdominal wall metastasis
5	65	M	1	III	SX2, IL-2, RX1	NED
6	69	M	0	III	SX4, RFAX2	Lung and liver metastasis

Abbreviations: Pt = patient; PS = performance status; NED = no evidence of disease; M = male; F = female; LN = lymph nodes; S = surgery; IFN-α = Interferon-α; IL-2 = Interleukin-2; Rx = radiation; RFA = radiofrequency ablation.

### Immunological response

**Figure 1:**
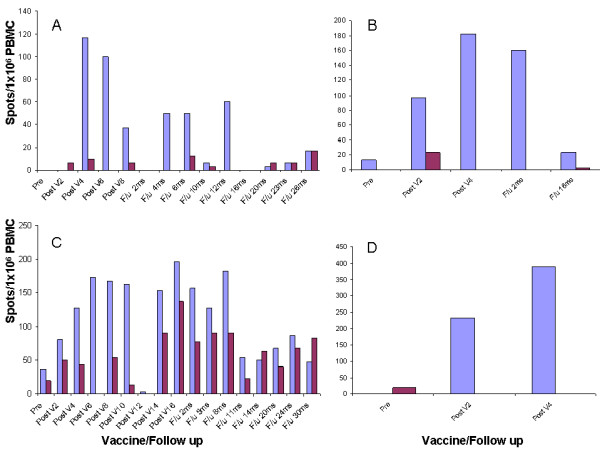
**Immune responses measured by ELISPOT assay**. ELISPOT results for all patients who had positive immune responses to the corresponding VHL peptide (spots/10^6 ^PBMC) in purple compared with the control peptide (TAX) in red: patient 2 (panel A); patient 6 (panel C); patient 3 (panel B); and patient 4 (panel D). Pre = prevaccination sample; Post V = postvaccination sample marked by the vaccine number; and F/u = follow up sample marked in months (ms) from the last post vaccine sample.

Patient 1 was excluded from immune analysis because of disease progression after only two vaccinations. Four out of the five evaluated patients (patients 2, 3, 4, and 6; 80%) generated specific immune responses against the corresponding mutant VHL peptides (Table 4). Patient 2 had no evidence of IFN-γ ELISPOT-reactive T cells prior to the vaccination; however, the frequency of these T cells increased dramatically after the fourth and six vaccinations to 117 and 100 spots/10^6 ^PBMC, respectively, compared with no response against the control peptide (TAX), and remained fairly elevated (50-60 spots) during the first 12 months of follow-up and then decreased dramatically (Figure 1A). Patient 6 had a similar immune response, having a significant increase in the number of IFN-γ ELISPOT-reactive T cells from 37 spots/10^6 ^PBMCs at baseline up to 163 spots/10^6 ^PBMCs after 10 cycles of vaccination and maintaining the immune response during the first 8 months of follow up (183 spots/10^6 ^PBMCs) before returning to baseline (Figure 1C).

**Table 4:** Clinical and immunological outcome

Patient	Cycles received	Off-therapy reason	Off-study status	PFS	OS	Immune response
1	2	P	P	2	17	Neg
2	10	PSC	NED	88 +	88 **+**	Pos
3	6	R	R	6.5	87 **+**	Pos
4	4	P	P	4	8	Pos
5	11	PSC	R	13.5	30.5	Neg
6	18	PSC	S	57 **+**	57 +	Pos

Abbreviations: P = progressive disease; R = recurrent disease; S = stable disease; NED = no evidence of disease; PSC = peptide stock completed; PFS = progression-free survival in months; OS = overall survival in months (both PFS and OS were calculated from the on-study date until progression, death, or last known follow-up marked as (+); Pos = positive immune response; Neg = negative immune response.

The six and four vaccinations that patients 3 and 4 received, respectively, were associated with an increase in the IFN-γ ELISPOT-reactive T cells, as shown in Figure 1B-1D. Patient 3 had a significant immune response after the fourth vaccination (from 13 spots/10^6 ^PBMCs at baseline up to 183 spots/10^6 ^PBMC); however, despite maintaining the immune response during the first 2 months of follow-up (160 spots/10^6 ^PBMCs), the number of reactive T cells then returned to baseline (Figure 1B). The number of IFN-γ ELISPOT-reactive T cells in patient 4 increased after the second and fourth vaccinations (from 0 at baseline up to 233/10^6 ^PBMCs and 390/10^6 ^PBMCs, respectively); however, this patient was lost to follow-up for additional immune endpoints (Figure 1D).

### Regulatory T cells (T regs)

**Figure 2:**
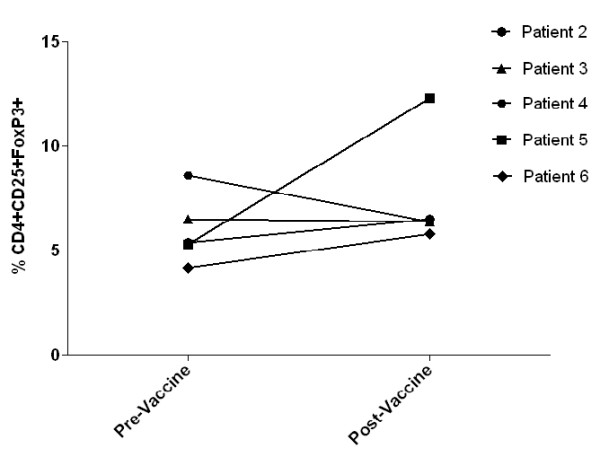
**Regulatory T cells (T regs)**. The percentage of T regulatory cells (CD4^+^CD25^+^FoxP3^+^) measured in the peripheral blood of the evaluable patients (patient 2, 3, 4, 5, and 6) pre and postvaccination. The postvaccination samples were taken during the last vaccination visit for every patient except patient 3 whom the last available T regs sample was during vaccination 5.

T regulatory cells (CD4^+^CD25^+^FoxP3^+^) were measured in the peripheral blood of the five evaluable patients (patients 2, 3, 4, 5, and 6) prevaccination and following each vaccination (Figure 2). No difference was found in the T regulatory cells frequencies in the postvaccination samples compared with prevaccination in four patients who demonstrated an immune response (patients 2, 3, 4, and 6). On the other hand, patient 5 who had no immune response to the corresponding peptide had a significant elevation in T regulatory cells in the post vaccination samples.

### Safety and toxicity

The vaccine was well tolerated. No grade III or IV toxicity occurred. The most common systemic adverse events were grade I and II fatigue (83% of patients) and local skin reaction in the form of mild skin redness and swelling (83% of patients), which resolved in less than 72 hours. No signs or symptoms of autoimmune disease were observed up to 88 months of follow-up.

### Clinical response

Patients received a total of 51 vaccinations. One of the treated patients did not complete the first four vaccinations (patient 1). This patient had extensive lung metastases and was removed from the study after two vaccinations because of rapid deterioration of performance status and disease progression. The other five patients received at least four vaccinations. Patient 3 had recurrent disease after six vaccinations. It is noteworthy that this patient underwent right adrenalectomy followed by subcarinal node resection and remained without any recurrence 87 months after enrollment on the study despite having no further therapy. Patient 4 was removed from the study after four vaccinations due to disease progression. The other three patients (patients 2, 5, and 6) received 10, 11, and 18 vaccinations, respectively, until the peptide stock was exhausted. Patients 2 and 6 completed the study and remained without disease recurrence (patient 2) or progression (patient 6) for 88, and 57 months, respectively, after starting on the study; both patients had no further conventional therapy after finishing the study. Patient 5 had recurrent disease during follow-up with cerebral metastases (Table 4). The median OS and median PFS for all six patients were 30.5 and 6.5 months, respectively.

## Discussion

The identification of the *VHL *gene and its critical role in renal malignancy has provided insight into the pathogenesis of sporadic clear cell renal carcinoma. It has also provided the potential for developing novel targeted therapies, including specific vaccines. In this pilot study we evaluated the feasibility of vaccination against mutant VHL peptides corresponding to the patients' own tumor mutations. We also tested the ability of this vaccine to generate a specific immune response against these mutations. The number of vaccinations varied among the six patients because it was dependent not only on the status of disease progression but also on the amount of the peptides available for use. We found that these custom-made mutant VHL peptide antigens were able to induce strong, specific immune responses detected by ELISPOT assay in four of the five evaluable vaccinated patients (80%). The immune responses of the three responding patients who had long-term follow-up share the same trend described as: 1) an increase in VHL peptide-specific T-cell frequency from baseline compared with the control peptide (TAX); 2) maintenance of the increased VHL-specific T-cell frequency throughout therapy; and 3) a return of the immune response to baseline after completion of the treatment.

Although cells other than T cells, such as NK cells and monocytes, present in PBMC utilized in the ELISPOT assays can secrete IFN-γ, the majority of IFN-γ secreting cells in the assays are T cells. Patients' autologous DCs were loaded with the specific peptides (10-17-mer VHL peptides) served as APC. Therefore, these peptides were presented in the appropriate context to stimulate T cell reactivity (MHC restricted peptides). Additionally, the number of IFN-γ secreting cells in response to the VHL-peptides increased after vaccination. This data demonstrates that the IFN-γ response measured in the ELISPOT is due to the induction of memory cells, and therefore T cells, to vaccination. As such, it is unlikely that any cells other than T cells are involved in the IFN-γ secretion. It would be interesting to distinguish between reactivity of CD8+ versus CD4+ T cells and if there are changes in these subsets, especially with those patients who demonstrated promising clinical out comes. However, for the purposes of this study, general T cell reactivity in response to vaccination was an appropriate measure to first assess if the vaccination could elicit an immune response to mutated self-antigen. Normally, the frequency of self-reactive T cells is quite low due to multiple mechanisms of central and peripheral tolerance. Findings from this pilot study demonstrate that we can elicit immunity to VHL peptides and thus provides the foundation for future studies to elucidate the particular immune responses generated by this vaccine.

Some cancer vaccine trials showed an increase of T regulatory cells which may be due to the progressive disease status or the use of certain cytokines, such as IL-2 [43,44]. Here we found that there was no increase in T regulatory cells in the postvaccination samples compared with prevaccination in all patients who demonstrated an immune response. The increase in T regulatory cells might have contributed to the limited efficacy of the vaccine in the only patient who failed to demonstrate an immune response. This also may indicate that the simple vaccination with antigens and adjuvants without cytokines may contribute less to the generation of T regulatory cells.

Vaccinating with mutant VHL peptides was found to be generally safe. The toxicities were all grade I or II and resolved spontaneously. This was a small pilot trial and was not powered to test the vaccine for clinical efficacy; however, despite the advanced disease status of these patients, we found that their median OS and median PFS were 30.5 and 6.5 months, respectively. Three of the six vaccinated patients are still alive (57, 87, and 88 months after starting on the trial) despite having no further conventional therapy, which is extremely unusual for patients with advanced RCC; interestingly, all three patients had a positive immune response to the corresponding peptide.

## Conclusions

In conclusion, we believe that vaccination with mutant VHL peptides is safe and effective in generating a specific immune response to the corresponding peptides. Manufacturing these custom-made peptides is time-consuming since it takes a cumulative 6-9 months to sequence the gene, manufacture the peptide, package it in vials, and conduct the appropriate required stability testing. This may pose practicality challenges in using such vaccination methods in advanced disease, considering the short life expectancy. Furthermore, as we have seen in this trial, the immune responses induced by these peptides along with adjuvant administered subcutaneously--as easy and practical as they may be--reverse gradually as soon as vaccinations are completed. Accordingly, we believe that such treatment needs to be continued in order to maintain meaningful immune response or use certain cytokines that can prolong the immune response such as IL-15 or GM-CSF [45,46]. That having been said, targeting *VHL *still provides a unique opportunity for a specific vaccine against RCC, especially in early disease, since there are very few known antigens in RCC. This trial draws attention to a novel therapeutic approach in RCC treatment that needs to be investigated further in larger clinical trials.

## Competing interests

The authors declare that they have no competing interests.

## Authors' contributions

OER	analyzed the data and drafted the manuscript

EA	participated in the patients care

RI	carried out the immunoassays

AT	carried out the immunoassays

BG	participated in the patients care

VEH	participated in the patients care

WML	analyzed the mutations

SMS	performed the statistical analysis

FG	provided the pharmaceutical support

GG	vialed the peptides and tested their stability

SAB	participated in the patients care

JAB	participated in the design of the study

SNK	conceived of the study, and participated in its design and coordination

All authors read and approved the final manuscript.
